# Doctors and Midwives

**Published:** 1904-01-16

**Authors:** 


### The Hospital.

A Journal of

The Medical Sciences and Hospital Administration.

Vol. XXXV.—No. 903. JANUARY 16, 1904.

### Doctors and Midwives.

We have received and publish on another page a communication on the part of the British Gynæcological Society, with reference to the relations likely to exist between medical practitioners and registered midwives, the general tone of which is, we think, much to be regretted. The Society desires to emphasise the distinction between women who wish to work as independent practitioners under the Midwives Board, and "for the most part amongst the very poor," and those who desire to do maternity nursing under the supervision of medical practitioners, and "for the most part amongst the middle and upper classes." The communication declares that the former class will act as competitors with medical men, and will not be likely to receive any assistance or work as monthly nurses from medical practitioners. The Society, in taking this ground, appears to us to be endeavouring to create rivalries which would have very little tendency to come into spontaneous existence, and to be doing its best, whatever that may be, to neutralise the advantages which an improved training of maternity nurses is calculated to confer upon the medical profession. We believe that general practitioners, as a rule, would be very glad to be relieved entirely from attendance in childbirth upon the "very poor," and that they would be equally glad to avail themselves of the services, as maternity nurses, of women in whose hands they might safely leave the business of watching over the earlier stages of labour, and whose competence for the purpose was not only known to themselves, but so certified by a public authority as to justify both doctors and patients in relying upon it in time of need.

It would be very difficult to estimate the amount of dislocation of work, in country practice, which is occasioned by the conditions under which attendance on midwifery cases has now to be conducted. A doctor who lives in a provincial town with a surrounding agricultural population, and whose area of work may extend five or six miles in every direction, will have a constant succession of midwifery cases at considerable distances from his home, and will be compelled for a time to neglect all other calls in order to attend to them. The causes of delay in natural labour, such, for instance, as slow dilatation of the os uteri, are, we think, of much more frequent occurrence in rural than in urban districts, and the course of parturition is more frequently protracted in the former than in the latter. In a town, moreover, the comparatively short distances between the homes of different patients can be quickly traversed, and the doctor who is in attendance upon a midwifery case can generally leave it, from time to time, for a period at least sufficiently long to enable him to attend to any matter of pressing urgency elsewhere. In the country the condition of things is wholly different. Patients, as a rule, are perfectly ready to admit the validity of the reason which the doctor's servants have to assign for any delay in his attendance—the reason that he is "at a labour"—but their readiness does not in the least degree serve to supply his place. It does not give the hypodermic injection to the patient who is in pain, or the timely dose of antitoxin at the commencement of diphtheria, or meet any one of the constantly recurring conditions in which promptitude of action is of the first importance. In the not uncommon occurrence of an emergency which is recognised as such by the friends of the patient, the detention of the family doctor at a labour case five miles off is often felt to be a sufficient reason for calling in the assistance of another practitioner, who is much more likely than a registered midwife to be a rival in practice, and whose intervention, however necessary, is always a source of possible difficulties and complications in the future. To put the matter in its simplest form, it may be said that the conditions of ordinary medical and surgical practice require regularity and punctuality of visiting, and ready accessibility in the case of unforeseen calls of an imperative character, while the claims of midwifery are often wholly antagonistic to these requirements. The employment as a maternity nurse of a woman who was also a registered midwife would be of the greatest possible assistance to the country doctor. It would enable him to visit the lying-in woman, to ascertain the absence of complications, to leave her for a specified time in hands that would not only be safe, but that would be recognised as safe by public opinion, and to return at reasonable intervals without anxiety as to what might be happening in his absence. Above all, it would enable him to do this without fear of being supplanted by a competing neighbour. We believe that hundreds of country practitioners would be extremely glad to get rid of midwifery practice altogether, and that they undertake it, not for the sake of the moderate honoraria which it produces, but almost exclusively because it serves to secure their professional position in the family. To relieve them of the burden without imperilling the position would in most cases be to confer upon them an almost unmixed advantage, and we are convinced that the education and registration of midwives ought to be conducive to the attainment of this end. The two classes, the doctors and the midwives, should be complementary, not antagonistic to one another; and those who attempt to encourage or to establish the latter condition are, we think, enemies to both classes alike. The only wise or patriotic endeavour would be to seek to establish complete harmony between them, of such a character that the doctor should largely avail himself of the assistance which trained midwives could render towards the orderly and methodical conduct of practice; while the midwives should be encouraged to feel that the doctors of the locality were their best friends, and should at all times be prompt to secure their aid when parturition was either attended by abnormality or followed by indications of disease.

